# Supplementary material for: Complexity and diversity in sparse code priors improve receptive field characterization of Macaque V1 neurons
Source: PLoS Comput Biol. 2021 Oct 25;17(10):e1009528. doi: 10.1371/journal.pcbi.1009528 (PMC8589190; doi:10.1371/journal.pcbi.1009528)
Supplement: S1 File — (PDF) [file pcbi.1009528.s001.pdf]

S1 File: Supplementary Materials

1 Responses characteristics of non-selected neurons

Neurons with responses above 0.2 to less than 50 stimuli in the dataset were discarded and not used for modeling. In the main paper we claim that the discarded neurons tend to have low responses to all stimuli, rather than being very sharply tuned. In Fig 1, we plot a histogram for each monkey of all its neurons' average response to their top 5 stimuli, a measure of their peak response, split by inclusion for modeling. The non-modeled neurons in blue, which do not pass the threshold of 50 $df/f > 0.2$ responses, clearly tend to have substantially lower responses than the modeled neurons. Thus, the neurons we discarded tend not to have very high response peaks. However, we found that when all the cells are used, the relative performance of all the models, and thus the conclusion of the paper remain the same.

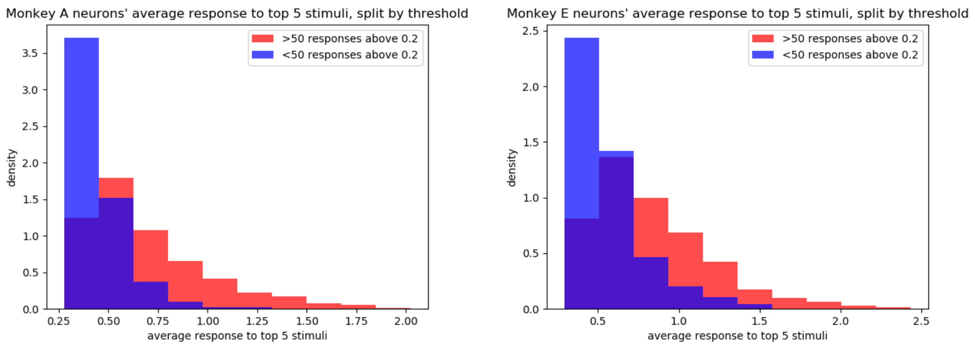

Fig 1. Histograms of top response values: For each monkey, we plot a histogram of its neurons' average response to the top 5 stimuli. We color the populations above (red) and below (blue) the threshold used for inclusion the main modeling results. Neurons below the threshold are much more likely to have a low response even to their most favored stimuli; that is, to not respond strongly to any stimuli. Note that the y-axis represents density—the count of below-threshold neurons is substantially lower than the count above threshold.

2 Frequency spectrum of GCNN and KFCNN kernels

The Gabor wavelet codes (used as GCNN kernels) and the complex-shaped sparse codes (used as FKCNN kernels) have similar coverage in the spatial frequency domain. We evaluate the similarity of the coverage by the percentage overlap of the two 2D power spectra, as well as the percentage in the overlap in the spectra in the frequency and the orientation dimensions.

The percentage overlap of the two power spectra is the ratio between the shared volume (Intersection) and the total volume (Union) of the two spectra. In 1D, it is the ratio between the intersection and the union of areas under the two curves. While the Gabor representation tiles the orientation and frequency domain more uniformly, the tiling of the complex-shape features will have some irregularity in the spectrum. The overlap of the 2D spectra is only 91%. However, when we average the power spectrum across orientation or spatial frequency, collapsing the 2-D spectrum into two sets of 1D spectra, we find the overlaps are 96% in the spatial frequency dimension and 95% in the orientation dimension, as shown in Fig 2. Thus, we consider the spectral coverage highly similar in both the spatial radial frequency and orientation dimensions.

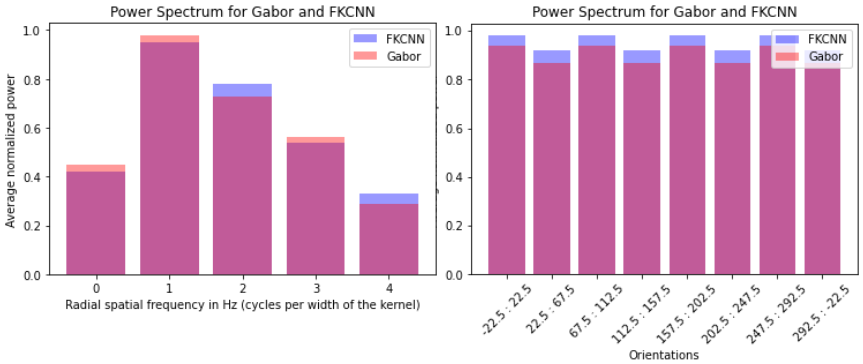

Fig 2. Frequency spectrum of GCNN kernels and FKCNN kernels. The image on the left shows the similarity between two model kernels' frequency spectrum along the radius distance dimension. The image on the left shows the similarity between two model kernels' frequency spectrum along the orientation dimension. The power on y-axis is first min-max normalized and then average.

3 Additional validation on synthetic data using pattern stimulus

In Section "Quantitative Evaluation of the Methods. Synthetic data validation" of the main paper, we test the validity of the improved methods using synthetic neurons and Gaussian white-noise images. To have more insight on these methods, we conduct the same experiment using synthetic neurons and the pattern stimulus.

Specifically, The synthetic neurons are modeled as the nonlinear transform of one or the sum of multiple linear filter outputs. We choose the ReLU activation function as our nonlinear transform because previous work found that all activation functions behave essentially the same [1]. Then, they are stimulated by the set of 9500 pattern images (20 x 20 pixels), and the response is given by the following linear-nonlinear model, as described by Equation 1. This is a common ground truth evaluation method, also used by [2, 3].

$$y_i = \sum_{s \in S} ReLU(I_i \cdot s) + \eta \tag{1}$$

where $\cdot$ denotes the inner dot product between the input image and the artificial RF s; $ReLU$ is the rectified linear unit function that maps negative values to 0; η represents Gaussian noise with zero mean and variance equal to $\frac{\sqrt{I_i \cdot s}}{10}$; the set of artificial RFs S is a subset of 9×9 filters padded with zeros in the surrounding to make them the same size as the input stimulus (20×20). We select S either from our learned sparse codes or cropped patches of our pattern stimuli. We train the models with 7000 samples of the pattern stimulus until convergence, 1500 stimulus as validation set for hyper-parameter tuning, and then test them with the rest 1000 stimulus.

The performance of the different methods in this validation experiment with pattern stimuli is shown in Fig 3. The patterns of behaviors are very similar to Fig 6 in the main paper, except the overall performance level is reduced. The performance of the methods using pattern stimuli might impose an upper bound performance that a model can achieve on this set of stimuli. The performance of the methods on V1 neurons of monkey E (shown in Fig 3) using this set of pattern stimuli is actually very close to this bound. This might suggest that the models are already doing the best they can on this set of stimuli.

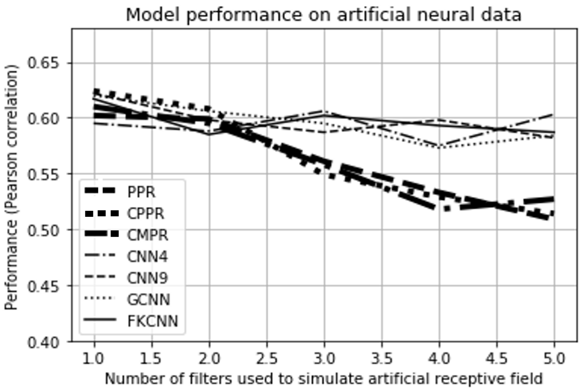

Fig 3. Model performance on artificial dataset with pattern stimulus

To evaluate whether the pattern stimuli can bias inferred RF subcomponents more than Gaussian white noise, we conducted the following additional experiments for CMPR and FKCNN. We used each of the 24 sparse coding kernels as the ground-truth RF of artificial neurons and compare how well CMPR and FKCNN can recover this kernel using the pattern stimuli versus the Gaussian white-noise stimuli. We run this experiment 50 times with different set of training stimuli (i.e. different seeding for random training set split). We found (1) CMPR can correctly select the true kernel 100% times using Gaussian white-noise stimuli but only 61.2% times using pattern stimuli. (2) For each FKCNN model, FKCNN found the correct kernel among the top five kernels based on importance 58.4% of the time, when Gaussian white noise was used, but 47.9% of the time when pattern data are used (shown in Fig 4). Taken together, the pattern stimuli can bias inferred RF subcomponents more than the Gaussian white noise. The bias due to pattern data might be very important for CMPR but less important for FKCNN.

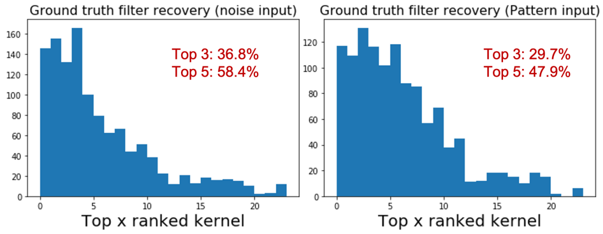

Fig 4. Comparing the consistency of how well FKCNN can recover receptive fields on white noise and pattern stimulus.

In addition, the subcomponents recovered by FKCNN should be considered as the basis that span the space of subcomponents that lead to the neurons' responses, but they are not unique. Nevertheless, they give us a glimpse of the stimulus patterns contribute to the cells' subcomponents.

These results reflect a key finding of Tang et al. (2018), that V1 neurons could be highly selective to some higher order features. They often did not respond to an oriented bar or grating, of any orientation, and their response to white noise stimuli tend to be poor. That was the rationale of using 9500 plus pattern stimuli—the hypothesis was to test whether V1 neurons are selective to some specific higher order local features, rather than being simple edge detectors. FKCNN or CMPR, regardless of whether white noise or pattern stimuli are used, are limited in that they tend to recover the general preference of the neurons rather than the local patterns they truly love.

Subsequent to our study, an iterative technique called the inception loop [4] has been developed to probe the optimal tuning of V1 neurons in mice and found comparable complexity in tuning.

4 HO neurons' preferred stimuli

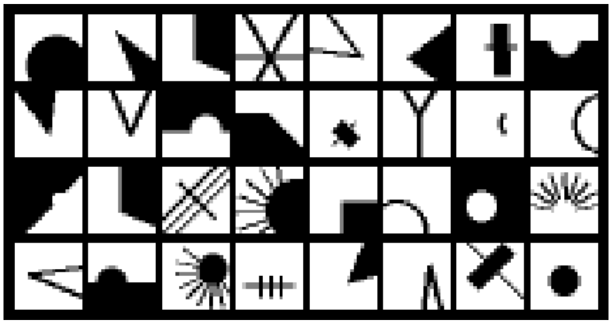

Fig 5. For 32 example neurons classified as having higher-order tuning, the most preferred stimulus.

Fig 5 shows the top stimuli for 32 example V1 neurons classified as HO by the method described in the paper (previously, in [1, 5]). They are more complicated than simple oriented bars, and can be compared to the filters obtained by convolutional sparse coding in Fig 2 of the main paper, with similar features like corners, curves, and crossing lines.

5 Alternative model front-ends

In order to further assess the suitability of the overcomplete convolutional sparse code basis for use in FKCNNs, we tested two other candidate bases, in additional to it and the Gabor wavelet bases. These are a set of Gabor-like filters obtained through classic sparse coding [6], and a set of filters from the object recognition-trained AlexNet CNN [7]. The AlexNet filters are averaged across the three color channels of the original model. The full set of 64 filters for each alternative basis is shown in Fig 6, but to in our models we only used 24 of these kernels for each basis (randomly sampled, with similar performance across multiple samples). The performance of each frontend, including the ones in the main paper, for each monkey is shown in Table 1.

	**Complex-Shaped**	**Gabor wavelets**	**Gaborlike Sparse Codes**	**AlexNet**
**Monkey A**	0.453	0.377	0.372	0.378
**Monkey E**	0.590	0.484	0.500	0.518

Table 1. Average CNN model performance with different frontends.

The convolutional sparse code basis continues to outperform the new ones, with the differences shown being significant at the $\alpha = 0.05$ level (marked as "+" in Table 1 of the main paper). This supports our claims that this basis is particularly good for predicting neural responses, as it outperforms these other reasonable candidate bases, though we cannot claim that no better basis exists.

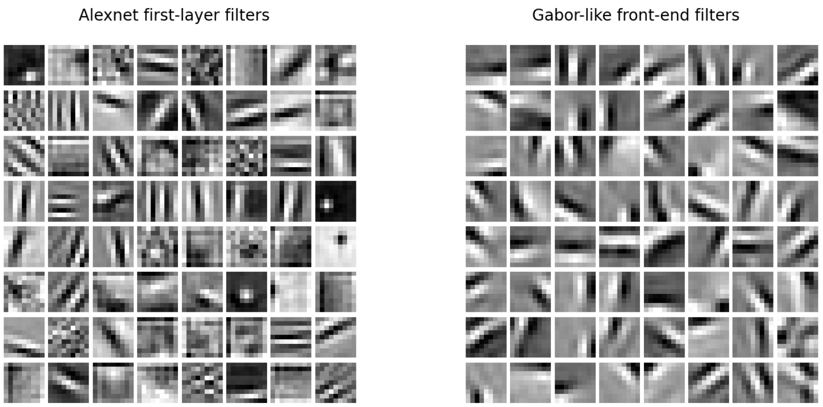

Fig 6. The filters of the AlexNet (left) and Gabor-like (right) alternative FKCNN bases.

6 Natural stimulus dataset

6.1 Receptive field characterization using a natural image dataset

The pattern stimulus set used in this study is biased in two aspects: First, it contains higher-order features at a higher rate than they would normally occur in natural images, even though it also contains a large number (1600) of oriented lines, edges and grating of different length, width, end-point, orientation parameters. Second, it is possible that certain features in natural scenes that neurons care about might not be in the stimulus set. Neurons coding for that features might not respond well at all. To assess the impact of these biases, particularly the first one, on receptive field characterization by our methods, we examined a set of neurons in one of the monkeys (Monkey A) that were also tested with 2250 natural images, each presented in a 4 DVA square aperture over the receptive fields which are typically 0.5-1.0 DVA in size, as reported in another paper [8]. The two experiments (pattern vs natural images) were performed a week apart apart, but 364 neurons can still be associated across the twp data sets, based on anatomical landmarks in the imaging data, and meet the response requirements on the pattern stimuli described in the main paper (primarily, at least 50 stimuli with a trial-averaged response greater than 0.2). For direct and fair comparison, we trained exactly the same FKCNN, GCNN, and CMPR models on these neurons' responses to the natural stimuli in the same way and with exactly the same models as described in the main paper for the pattern stimuli, and evaluate two quantitative metrics concerning HO and OT neurons characterization.

**Model**	**Performance**	
	Pattern stimuli	Natural image stimuli
FKCNN	0.453	0.215
GCNN	0.377	0.197

Table 2. Predictive performance of each CNN kernel set on the natural image stimuli and pattern stimuli for Monkey A. Performance is substantially higher for the responses to pattern stimuli.

The predictive performance of the FKCNN and GCNN for natural images is significantly lower for this set of data than for the artificial stimuli, as shown in Table 2. This could be due to many factors: (1) natural stimuli are more complex; (2) each

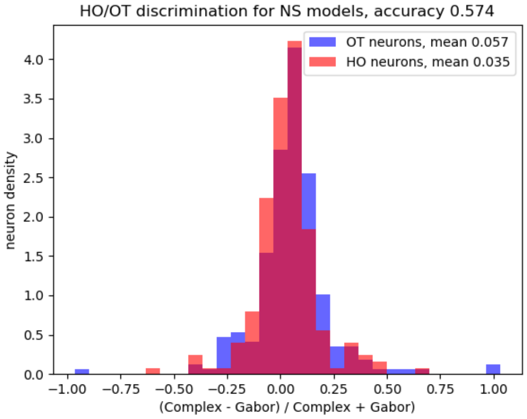

Fig 7. Histogram of FKCNN-GCNN score differences for HO and OT neurons with models trained on the natural stimuli. Compared to the results on the pattern data in Fig 10 of the main paper, the separability of the cell classes is visibly lower. Quantitatively, the linear separability of the cell classes is 57%, essentially identical to chance (also 57%) on this subset of neurons.

stimulus was only tested in 3 trials, leading to a noisier average response; (3) stimulus size is at least 4 times larger than the receptive field and thus nonlinear contextual surround effects could contribute to the high sparsity in the neurons' response, etc. Given the complexity of natural images, a deeper network is usually required for natural images. However, for this dataset, because of the sparsity in response and data size, even a deeper network does not yield considerable better results. Nevertheless, one encouraging observation is that the predictive performance of the FKCNN is 10% better than GCNN (0.215 versus 0.197), a similar, but smaller gap than the models trained on the pattern stimuli (10% compared to 15% relative to the GCNN). This supports our main claim made based on the artificial data.

Secondly, we also compared the top kernels yielded by the CMPR method. While OT kernels are recovered as the top kernels for a majority of the OT neurons for both sets of stimuli, OT kernels are selected as the top kernels for only 31.4% of the HO neurons based on pattern stimuli, but 78.7% of the same neurons based on natural image stimuli, representing a 150% relative increase. This suggests that the CMPR method trained on natural stimuli tends to identify simple oriented edge filters as the top kernels even for HO neurons, in contrast to its behavior on the pattern stimuli. As we discussed in the paper, the kernels recovered by the FKCNN are not unique—different subsets of the dictionary, and different combination of the kernels of the same subsets, or even "noisy" kernels recovered by standard CNNs can be used to compose a receptive field model to yield reasonable prediction performance. This is because the kernels are simply a basis for spanning the stimulus-response space of a receptive field. Characterizing and comparing the stimulus-response manifolds is difficult that requires considerable further research effort. We can visualize the receptive fields of the neurons based on pattern stimuli as done in [1] for the baseline CNN, but we fail to visualize the receptive fields of the models based on natural image data, likely because of the neural responses to the natural images are too sparse, resulting in poor regression-based models.

Thirdly, we evaluated the FKCNN-based metric (differences between FKCNN and GCNN model performance) for the OT and HO populations based on natural images. While Fig 10 of the main paper, based on pattern stimuli, shows a clear distinction in

this metric between the two groups of neurons, Fig 7 here shows that HO and OT neurons cannot be distinguished based on this metric derived from the responses to natural stimuli.

It is important to note that we are not claiming artificial stimuli are better than natural stimuli for characterizing neurons. The artificial stimuli could be better than natural images for probing receptive fields only when data are limited. When the data available in the natural image data-set are sufficiently large, we expect natural image should provide more complete and accurate characterization of the neurons' transfer functions, particularly when used with a deeper network.

References

1. Zhang Y, Lee TS, Li M, Liu F, Tang S. Convolutional neural network models of V1 responses to complex patterns. Journal of Computational Neuroscience. 2018; p. 296301.

2. Rust NC, Schwartz O, Movshon JA, Simoncelli EP. Spatiotemporal Elements of Macaque V1 Receptive Fields. Neuron. 2005; p. 945–956.

3. Klindt DA, Ecker AS, Euler T, Bethge M. Neural system identification for large populations separating "what" and "where". In Advances in Neural Information Processing Systems 30. 2017;.

4. Walker EY, Sinz FH, Cobos E, Muhammad T, Froudarakis E, Fahey PG, et al. Inception loops discover what excites neurons most using deep predictive models. Nature neuroscience. 2019;22(12):2060–2065.

5. Tang S, Lee TS, Li M, Zhang Y, Xu Y, Liu F, et al. Complex Pattern Selectivity in Macaque Primary Visual Cortex Revealed by Large-Scale Two-Photon Imaging. Current Biology. 2018; p. 38–48.

6. Olshausen BA, Field DJ. Emergence of simple-cell receptive field properties by learning a sparse code for natural images. Nature. 1996;381:607–609.

7. Krizhevsky A, Sutskever I, Hinton GE. ImageNet Classification with Deep Convolutional Neural Networks. In: Proceedings of the 25th International Conference on Neural Information Processing Systems - Volume 1. NIPS'12. Red Hook, NY, USA: Curran Associates Inc.; 2012. p. 1097–1105.

8. Tang S, Zhang Y, Li Z, Li M, Liu F, Jiang H, et al. Large-scale two-photon imaging revealed super-sparse population codes in the V1 superficial layer of awake monkeys. eLife. 2018;7:e33370. doi:10.7554/eLife.33370.
